# Ultraviolet Functionalization of Electrospun Scaffolds to Activate Fibrous Runways for Targeting Cell Adhesion

**DOI:** 10.3389/fbioe.2019.00159

**Published:** 2019-06-26

**Authors:** André F. Girão, Paul Wieringa, Susana C. Pinto, Paula A. A. P. Marques, Silvestro Micera, Richard van Wezel, Maqsood Ahmed, Roman Truckenmueller, Lorenzo Moroni

**Affiliations:** ^1^Tissue Regeneration Department, MIRA Institute for Biomedical Technology, University of Twente, Enschede, Netherlands; ^2^Department of Mechanical Engineering, TEMA, University of Aveiro, Aveiro, Portugal; ^3^Complex Tissue Regeneration Department, MERLN Institute for Technology Inspired Regenerative Medicine, Maastricht University, Maastricht, Netherlands; ^4^BioRobotics Institute, Scuola Superiore Sant'Anna, Pisa, Italy; ^5^Translational Neural Engineering Laboratory, Center for Neuroprosthetics, School of Engineering, École Polytechnique Fédérale de Lausanne, Institute of Bioengineering, Lausanne, Switzerland; ^6^Biophysics, Donders Institute for Brain, Cognition and Behaviour, Radboud University, Nijmegen, Netherlands; ^7^Biomedical Signals and Systems, MedTech Center, University of Twente, Enschede, Netherlands

**Keywords:** tissue engineering, scaffold, electrospinning, UV irradiation, photopatterning, cell adhesion

## Abstract

A critical challenge in scaffold design for tissue engineering is recapitulating the complex biochemical patterns that regulate cell behavior *in vivo*. In this work, we report the adaptation of a standard sterilization methodology—UV irradiation—for patterning the surfaces of two complementary polymeric electrospun scaffolds with oxygen cues able to efficiently immobilize biomolecules. Independently of the different polymer chain length of poly(ethylene oxide terephthalate)/poly(butylene terephthalate) (PEOT/PBT) copolymers and PEOT/PBT ratio, it was possible to easily functionalize specific regions of the scaffolds by inducing an optimized and spatially controlled adsorption of proteins capable of boosting the adhesion and spreading of cells along the activated fibrous runways. By allowing an efficient design of cell attachment patterns without inducing any noticeable change on cell morphology nor on the integrity of the electrospun fibers, this procedure offers an affordable and resourceful approach to generate complex biochemical patterns that can decisively complement the functionality of the next generation of tissue engineering scaffolds.

## Introduction

Many tissue engineering (TE) strategies use scaffolds to recreate biochemical and biomechanical cues that modulate cell response with the final purpose of mimicking specific *in vivo* microenvironments (Minardi et al., 2016; Caddeo et al., 2017). While the dynamic relationship *in vivo* between cells and their surroundings also include neighboring cells and growth factors, it is the architecture and chemical composition of the ECM that spatially and temporally coordinate the activation of specific cell receptors and consequently the signaling cascades that impact gene expression, cell phenotype and, ultimately, cell fate (Li et al., 2018). Hence, the engineering of the next generation of TE scaffolds is currently focusing on reproducing the biochemical, mechanical and topographical properties of the targeted ECM in an effort to induce synergetic effects on cells that stimulate the production of new ECM at a rate compatible with the degradation of the used biomaterial (Li et al., 2017). Considering the fibrillar configuration of the majority of the ECM elements (Theocharis et al., 2016), nanofibrous scaffolds emerged during the past few years as viable biomimetic platforms to boost new tissue formation since their high area-to-volume ratio and adjustable porosity are capable of enhancing cell adhesion and function while facilitating nutrient diffusion and metabolic waste removal (Stocco et al., 2018). Thus far, electrospinning has consistently maintained interest to engineer these scaffolds as it successfully enables the production of uniform fibers with controlled diameter from a wide range of materials (Xue et al., 2017; Chen et al., 2018). Additionally, important topographical scaffolding features (Metavarayuth et al., 2016) like micropatterns (Zhong et al., 2015; Kang et al., 2018) and roughness (Chen et al., 2017a) can be easily adjusted by simply adapting the fabrication parameters of the electrospinning process with the final purpose of inducing specific cell behaviors concerning their adhesion, proliferation, migration and differentiation. The accuracy of the distribution of the biophysical cues can be further augmented by combining electrospinning with microcontact printing as it was reported by Li et al. (2016) who successfully transferred fibronectin lane patterns from a polydimethylsiloxane stamp to an electrospun mesh, leading to an efficient neural differentiation of stem cells without biochemical inducers (e.g., growth factors). However, the inclusion of biochemical cues is a mandatory requisite for generating cell-material interactions able to precisely simulate the biological activities of a specific living tissue, where the ECM presents gradients/patterns of biomolecules that are independent of the topographical variations (Benetti et al., 2016; Li et al., 2017).

In this way, standard approaches such as plasma treatment are considered suitable to spatially modulate the surface chemistry of electrospun scaffolds relatively to alternative methodologies that restrict the number of possible TE applications due to the use of uncommon polymers (Viswanathan et al., 2015) or increased technical complexity of the electrospinning setup (Kishan et al., 2017). Plasma treatment is an efficient and versatile functionalization technique since it allows the production of reactive species from simple gases (e.g., oxygen, nitrogen) capable of triggering specific chemical reactions onto the surface of the fibers and therefore generating different chemical groups without altering the bulk properties of the biomaterial (Duque Sánchez et al., 2016; Hetemi and Pinson, 2017). For example, it was reported that the introduction of oxygen and nitrogen functional groups onto the surface of electrospun scaffolds could lead to an enhanced proteins/biomolecules adsorption and consequently to a larger number of anchoring points for cells, influencing their attachment, spreading and shape (Qiu et al., 2017; Babaei et al., 2018). In this context, it is possible to shape linear (Tanes et al., 2017) and circular (Wu et al., 2018) chemical gradients by creating concentration and time dependent graded masks of inert bovine serum albumin (BSA) able to block the plasma induced chemical groups from selected zones, potentiating counter current functionalized regions of the selected bioactive agent. However, this surface modification strategy depends on a vacuum system and can often damage the electrospun fibers, especially following prolonged exposure that is often needed to improve its otherwise limited penetration depth (Hasan et al., 2014; Duque Sánchez et al., 2016).

As an alternative, affordable and well-known sterilization methods (e.g., gamma and ultraviolet (UV) irradiations) could be adapted to modify the surface chemistry while preserving the morphology and alignment of the electrospun fibers (Valente et al., 2016). Particularly, UV irradiation enables the production of a wide range of radical groups onto the surface of the electrospun fibers that can be further used in customized grafting reactions (e.g., crosslinking, covalent attachment) (Duque Sánchez et al., 2016; Hetemi and Pinson, 2017). Indeed, recent reports have pointed out that the surface modification of electrospun fibers via UV treatment is able to guarantee not only the efficacious anchoring of chemical guidance cues for enhancing cell adhesion and proliferation (Kador et al., 2014; Piai et al., 2017), but also the attenuation of specific limitations of biomaterials like water solubility since approaches like UV curing can be used to covalently crosslink two electrospun meshes with complementary hydrophilic/hydrophobic profiles at the molecular scale with the intention of guaranteeing a final insoluble polymer network (Bazbouz et al., 2018). Additionally, the introduction of complex chemical gradients can be engineered by spatially limiting the UV irradiation to targeted regions of the scaffold—photopatterning—and controlling the surface density of the bioactive agent via adjustments in the biomolecules/polymer concentration, UV light intensity and exposure time (Wade et al., 2015). Although UV photopatterning is being used in TE platforms with promising outcomes regarding the modulation of cell response (Han et al., 2015; Kim et al., 2015), results related to electrospun scaffolds are fairly confined to the creation of functionalized zones with detailed geometries able to immobilize biomolecules (Hersey et al., 2014; Kalaoglu-Altan et al., 2016).

Taking this into account, we investigated a simple and efficient UV photopatterning strategy able to expand the bioactivity of PolyActive™ (PA) electrospun scaffolds by introducing biochemical cues onto the surface of poly(ethylene oxide terephthalate)/poly(butylene terephthalate) (PEOT/PBT) fibers in a spatially accurate manner. Indeed, since the surface chemistry of the scaffolds changed with the UV treatment time, it was possible to precisely modulate the specific functional groups located onto the target areas, where the protein adsorption process was potentiated, and therefore a preferential cell attachment was instigated. Based on the obtained results, we propose that UV functionalization of electrospun scaffolds could be a suitable approach to induce efficient biochemical gradients toward an enhanced simulation of cellular microenvironments.

## Materials and Methods

### Materials

The PA copolymers with compositions of 300PEOT55PBT45 (PA 300) and 1000PEOT70PBT30 (PA 1000), where 300 and 1000 are the molecular weight in g mol^−1^ of the starting polyethylene glycol blocks used during the copolymerization and the ratios of the PEOT:PBT segments are 55:45 and 70:30, respectively, were purchased from PolyVation BV. Chloroform, 1,1,1,3,3,3-Hexafluoro-2-propanol (HFiP), fluorescein isothiocyanate conjugated bovine serum albumin (FITC-BSA), phosphatase buffered saline (PBS), Dulbecco's Modified Eagle Medium (DMEM), fetal bovine serum (FBS), penicillin-streptomycin (pen-strep), formalin, triton-X solution, and DAPI were purchased from Sigma-Aldrich. The Alexa Fluor 594 conjugated phalloidin was purchased from ABCAM. Rat Schwann cells (rSCs), cell line RT4-D6P2T, were purchased from ATCC.

### Scaffolds Fabrication

PA 300 and PA 1000 were firstly dissolved in mixtures of chloroform:HFiP with a ratio of 8:2 (v:v) and final concentrations of 20 and 15% w/v, respectively. Then, after stirring overnight at room temperature, both polymer solutions were electrospun through a 21G blunt-tip needle at a controlled flow rate (1 mL h^−1^) and using a gap electrode collector to induce fiber alignment. The distance between the two collector electrodes was 10 mm and the ambient conditions were kept constant (25°C and 35% humidity). For the electrospinning of PA 300, a voltage of 20 kV and a working distance of 200 mm were applied; on the other hand, PA 1000 electrospun fibers were fabricated by using a voltage of 25 kV and a distance between the spinneret and the collector of 250 mm. The thickness of both PA 300 and PA 1000 electrospun scaffolds were measured with a micrometer, presenting a final value of ~20 μm.

### Surface Functionalization

UV radiation was used to change the chemical composition of the surface of the PA 300 and PA 1000 electrospun scaffolds. Briefly, the scaffolds were positioned at a distance of 200 mm relatively to the source (UV Crosslinker, UltraLum CEX-800) with the purpose of irradiating the surfaces of the fibrous networks with a UV wavelength of 254 nm and energy of 1,200 mJ during 10, 40, and 90 min. Additionally, a nickel mask with outer dimensions of 250 × 250 mm was used to selectivity activate specific areas onto the surfaces of the scaffolds, leading to a spatially controlled patterning. More precisely, when the mask was in contact with the scaffolds, the UV irradiation has only interacted with the electrospun fibers through 140 opened parallel rectangles (190 mm high × 0.21 mm width) regularly spaced by 0.10 mm. Depending on the UV exposure time, the samples were named “PA 300 UVX” or “PA 1000 UVX”, where X is the time in minutes of the irradiation period.

### Scaffolds Characterization

Scanning electron microscopy (SEM) (Philips XL 30 ESEMFEM) was used to conduct the morphological characterization of both PA 300 and PA 1000 electrospun scaffolds. The fibers' diameter was determined from 10 SEM pictures (each picture containing a minimum of 30 fibers) per sample (*n* = 5) using ImageJ software. The effect of the UV irradiation onto the surface of the scaffolds was studied by analyzing the variations in the attenuated total reflectance Fourier transform infrared (ATR-FTIR) (Bruker ALPHA FT-IR Spectrometer) spectra for the selected timepoints (0, 10, 40, and 90 min). The ATR-FTIR spectra were recorded between 400 and 4,000 cm^−1^ at a resolution of 4 cm^−1^ and average of 64 scans. Indeed, the evolution of the areas beneath the ATR-FTIR peaks relatively to the UV exposure time were calculated and used as qualitative indicators of the gradual alteration of the chemical functional groups located onto the fibrous surfaces. The relative changes (%) were calculated by simply considering the ratio between the peak areas of the UV irradiated samples and the correspondingly original peak areas (non-irradiated samples). Moreover, the chemical elemental composition of the PA 300, PA 300 UV40, PA 1000, and PA 1000 UV40 scaffolds was further studied by X-ray photoelectron spectroscopy (XPS) [Quantera SXM (scanning XPS microprobe, Ie 2.6 mA, power 50 W, working pressure ~ 1.3 e-8 torr) from Physical Electronics] analysis. The XPS measurements were acquired on the electrospun scaffolds on at least four points per sample with a monochromatic Al Kα radiation at 1,486.6 eV. The C 1s main peak was set at 284.8 eV and XPSPEAK Version 4.1 software was used to analyze the data.

The static water droplet contact angles (Dataphysics OCA 20 contact angle system) of the PA 300, PA 300 UV40, PA 1000, and PA 1000 UV40 materials were measured at ambient temperature. Since this analysis was not accurate when using the electrospun scaffolds due to the immediate entrapment of the MilliQ water droplets into the fibrous networks, an equivalent set of flat polymeric films were fabricated by evaporation casting with the purpose of evaluating the wettability changes induced by the UV treatment without a fast outflow (Nandakumar et al., 2012; Di Luca et al., 2016). Briefly, 2 mL of PA 300 and PA 1000 electrospinning solutions were firstly placed in glass containers (15 mm of diameter) and then left to evaporate at room temperature. The resultant polymeric films were then carefully washed with MilliQ water and dried before a 40 min period of UV exposure under the same conditions as their fibrous counterparts. The contact angle was measured at five different spots on each sample (*n* = 3 per condition).

### Protein Adsorption Tests

FITC-BSA was selected as model protein for evaluating the capacity of the newly formed functional groups located onto the surface of the PA 300 UV40 and PA 1000 UV40 fibrous scaffolds to adsorb proteins. Firstly, both treated and untreated samples (*n* = 3 for each condition) were carefully washed with distilled water and PBS, then the scaffolds were incubated in a solution of FITC-BSA in PBS with a concentration of 10 μg mL^−1^ at room temperature for 2 h. After this period, the scaffolds were washed again with distilled water and PBS with the purpose of removing any non-adherent proteins. Further analysis was carried out by fluorescence microscopy observations (Nikon Eclipse e600).

### Cell Culture and Cell Adhesion Tests

Prior to cell culture, each PA 300, PA 1000, PA 300 UV40, and PA 1000 UV40 scaffold (*n* = 4 for each condition) covered an individual well of a non-treated 24 well-plate (Thermo Scientific™ Nunc™), where the samples were sequentially sterilized in 70% ethanol for a period of 2 h, rinsed with PBS and finally incubated in culture medium (DMEM + 10% FBS + 1% pen-strep) for 2 h with the purpose of allowing protein adhesion. This medium was then removed and 15 × 10^3^ rSCs suspended in 200 μL of culture medium were seeded in each scaffold. Cell attachment was enhanced by incubating the samples at 37°C in a humid atmosphere with 5% CO_2_ for 2 h, after which, fresh medium was added until a final volume of 2 mL per well was reached. The adhesion, morphology and spatial distribution of the rSCs on the scaffolds were analyzed after 12 h of incubation via fluorescence microscopy (Nikon Eclipse e600). Briefly, the samples were rinsed with PBS before the cells were firstly fixed in a 10% paraformaldehyde solution for 20 min and then permeabilized using 0.5% v/v Triton X for 10 min. Additionally, before the cytochemical staining of the rSCs, the samples were incubated in a 1% BSA solution for 25 min in order to reduce the non-specific background staining. Finally, the actin filaments and the nuclei of the rSCs were stained in a dark room during periods of 90 min (phalloidin) and 10 min (DAPI), correspondingly.

### Cell Elongation Tests

The elongation of the rSCs was analyzed by calculating the elongation factor E, which equals to the division of the longer axis of the cell divided by its smaller axis minus one (Stylianou et al., 2018). In detail, ImageJ software was used to calculate E from fluorescence pictures where it was possible to accurate discern individual rSCs (*n* > 30 per sample).

### Statistical Analysis

Statistically significant differences were determined by using a one-way analysis of variance (ANOVA) followed by Tukey's multiple comparison test (Origin Software, ^*^p < 0.05). Data are expressed as mean ± standard deviation.

## Results and Discussion

**Figure 1:**
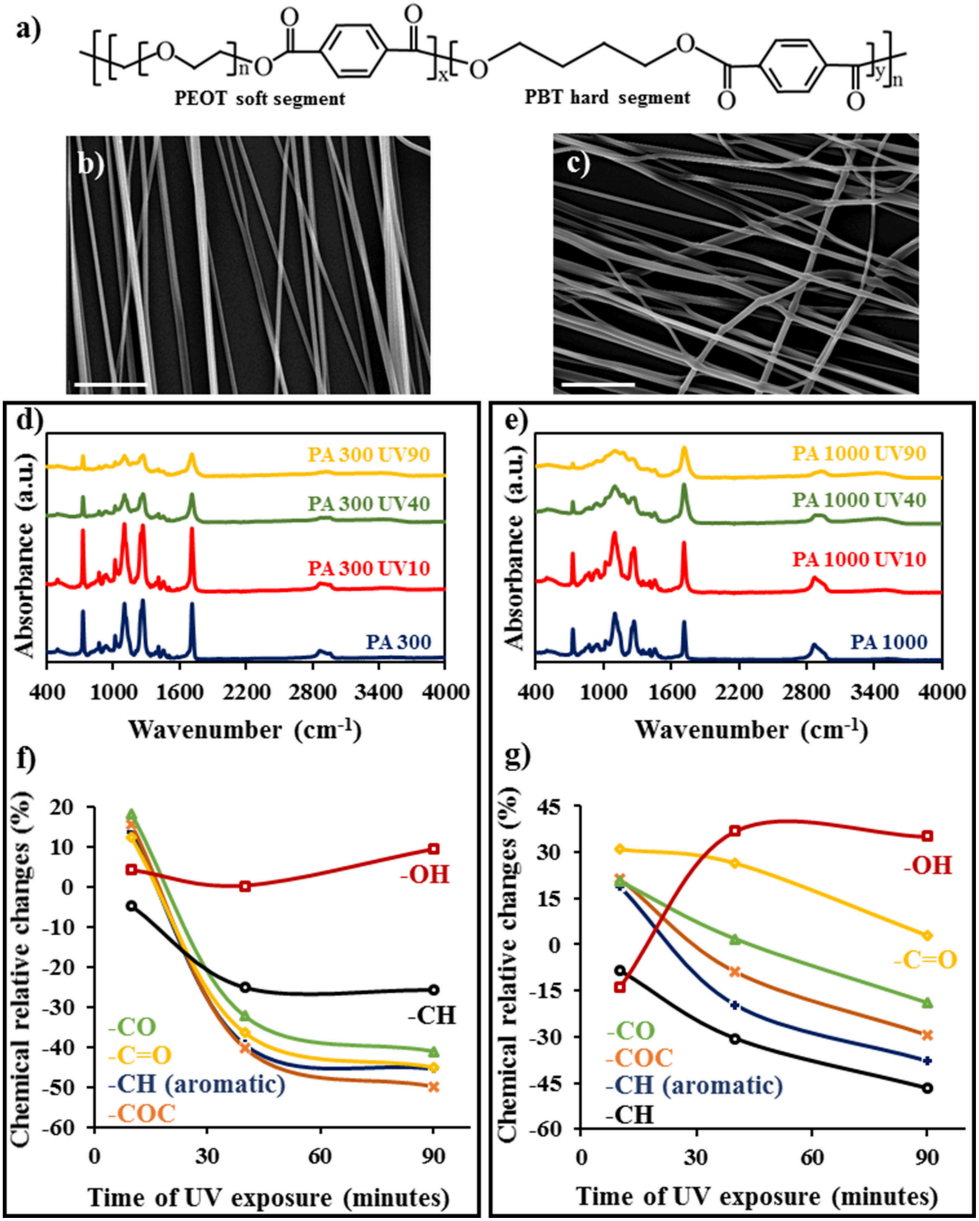
Surface chemical modification of PA scaffolds. **(a)** Chemical structure of PA block copolymers; Morphological properties of the untreated PA 300 **(b)** and PA 1000 **(c)** electrospun fibers; ATR-FTIR spectra of the UV functionalized PA 300 **(d)** and PA 1000 **(e)** electrospun fibers at several time points; changes in the peaks intensity of UV functionalized PA 300 **(f)** and PA 1000 **(g)** scaffolds relatively to the untreated samples at several time points. Scale bars = 10 μm.

PA (Figure 1a) was chosen as bulk material since it is a well-studied biocompatible copolymer composed of amorphous, hydrophilic PEOT segments intercalated with crystalline, hydrophobic PBT blocks. PA has been successfully used in a wide range of TE applications, including bone (Nandakumar et al., 2010), cartilage (Chen et al., 2017b), and neural (Santos et al., 2017) regeneration approaches. Indeed, the balancing between the soft elastomeric, hydrogel-like behavior in the soft segments and the rigidity introduced by the hard segments can be modulated by adapting the length and/or the ratio of the PEOT/PBT blocks, leading to tunable biological, mechanical and degradation performances. This remarkable adaptation to different strategies was already recognized by the US Food and Drug Administration, which approved the use of PA as bone cement restrictors and tympanic membrane reconstruction biomaterials (Kutikov and Song, 2015). From the PA copolymer family, PA 300 and PA 1000 were selected because of their ability to encourage excellent *in vitro* and *in vivo* performances while inducing complementary cell behaviors (Jansen et al., 2009; Hendriks et al., 2013).

As expected (Yixiang et al., 2008; Valente et al., 2016), the morphological and topographical features of the electrospun scaffolds remained unchanged for the selected UV irradiation periods (10, 40, and 90 min). SEM analysis revealed not only smooth and defect free PA 300 and PA 1000 fibers with average diameters of 0.50 ± 0.10 μm and 0.70 ± 0.28 μm, respectively, but also a preferential alignment of both fibrous networks due to the successful modulation of the electric field during the electrospinning process by the parallel electrodes (Li et al., 2003) (Figures 1b,c). On the other hand, the UV treatment induced noticeable time-dependent chemical modifications onto the surfaces of the PA 300 and PA 1000 electrospun scaffolds that were studied by analyzing the variations of their ATR-FTIR spectra (Figures 1d,e). Relatively to the untreated samples, both fibrous systems displayed standard absorbance bands associated with the functional groups present on the soft and hard segments (Gong and Parsons, 2012; Karunakaran et al., 2015), showing intense peaks associated with the aromatic C-H out of plane bending at 730 cm^−1^, the C-O-C stretching of the ether located at 1,102 cm^−1^ and the C-O and C = O stretch modes of the ester positioned at 1,270 and 1,714 cm^−1^, respectively. Moreover, it is possible to identify an absorbance region related with the vibrational stretch of the C-H_2_ (2,840–3,000 cm^−1^), where the characteristic peaks of the symmetric (2,860 cm^−1^ for PA 300 and 2,869 cm^−1^ for PA 1000) and asymmetric (2,950 cm^−1^ for PA 300 and 2,960 cm^−1^ for PA 1000) stretching features are perceptible. For each time point, the modifications registered in the ATR-FTIR spectra correspond to competition between scission chain events and the formation of new oxygen moieties due to the several stages of the UV induced photodegradation, which leads consequently to the growth of a new broad region between 3,100 and 3,700 cm^−1^ correlated with the O-H stretch mode. The increase of this absorbance band is particularly prevalent after the first 40 min of irradiation onto the PA 1000 fibers since, contrary to the PA 300 sample, its higher PEOT/PBT ratio and longer PEO polymer chain boosted the generation of hydroxyl and carboxyl functionalities; the presence of these groups was also confirmed by the emergence of a new peak associated with the C-OH stretching (1,164 cm^−1^) (Oomens et al., 2009) in the PA 1000 UV40 and PA 1000 UV90 ATR-FTIR spectra. In fact, the effects of the PEOT photooxidation mechanisms (Morlat and Gardette, 2001) were substantially more noticed in the PA 1000 polymer chain during the UV treatment as it is possible to observe by the remarkable decrease of the intensity of the C-H_2_ stretching region. In contrast, the more crystalline PA 300 underwent hydrogen abstraction reactions onto the hard PBT segments which generated aromatic mono and di-hydroxy-substituted compounds together with other oxidation products such as carboxylic acids and derivatives of esters (Grossetête et al., 2000; Gardette et al., 2014). The formation of ester functionalities was also observed after 10 min of UV irradiation for both PA 300 and PA 1000 due to β-scission of the alkoxy radicals generated from the PEOT segment and the reactions between radicals under thermo-oxidative conditions, contributing to the intensification of the absorbance peaks located at 1,714, 1,270, and 1,102 cm^−1^. The subsequent decrease of these peaks is related to the bigger number of chain scission and crosslinking events that occur under continuous UV exposure. A similar behavior can be observed for the absorbance band at 730 cm^−1^ since its intensity firstly increased due to the out of plane bending of the produced O-H functional groups and then diminished because of the substantial degradation of the aromatic rings. Taken together, these results (Figures 1f,g) revealed that PA 300 UV40 and PA 1000 UV40 electrospun scaffolds achieved a balance of increased functional groups known to efficiently interact with proteins while exhibiting limited degradation and were therefore selected for further cell adhesion testing.

**Figure 2:**
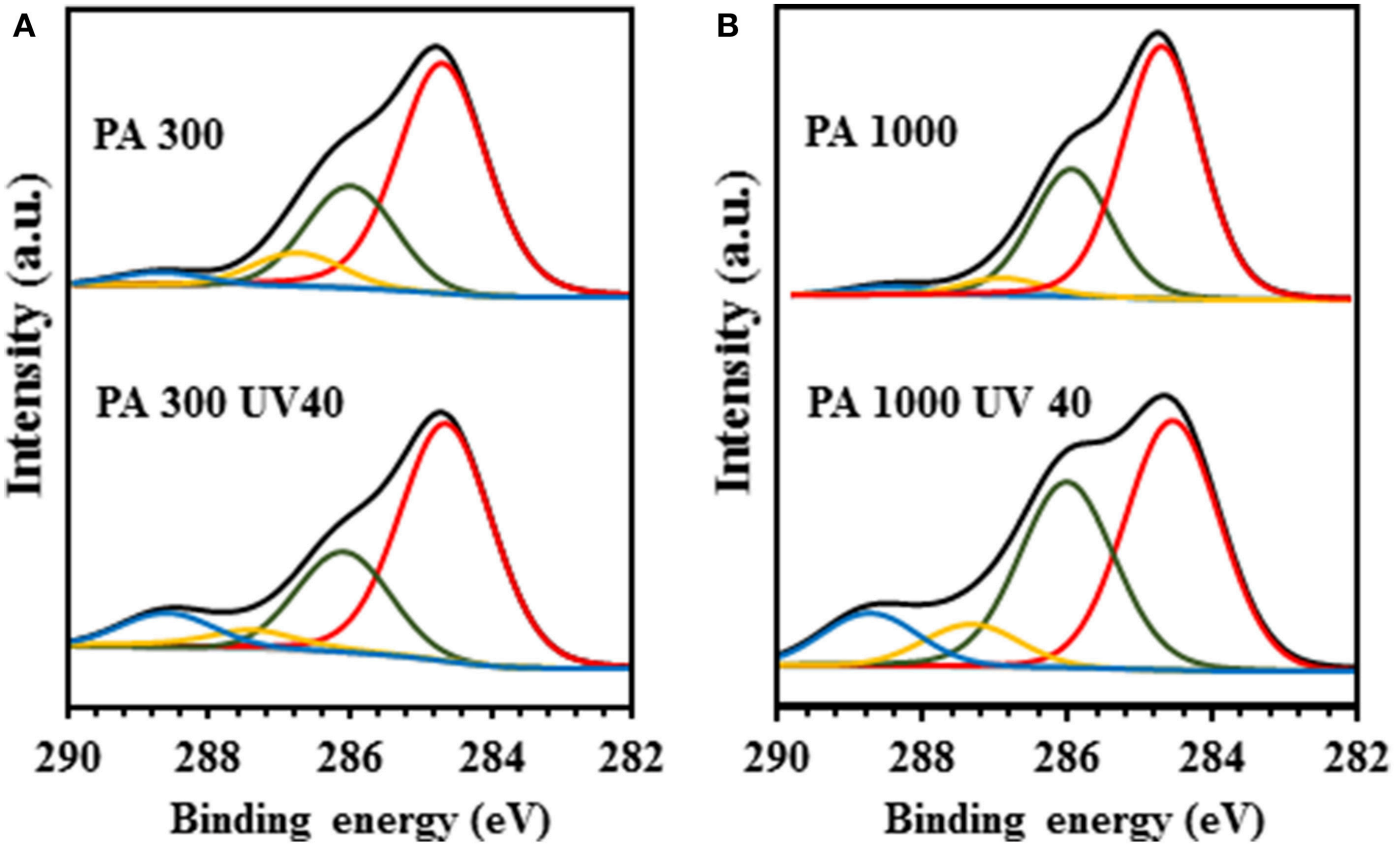
XPS analysis of the PA scaffolds. **(A)** C 1s peaks of the PA 300 and PA 300 UV40 electrospun fibers and **(B)** C 1s peaks of the PA 1000 and PA 1000 UV40 electrospun fibers.

The XPS analysis of the untreated and UV40 electrospun scaffolds confirmed the ATR-FTIR results, showing the expected C 1s region (Nandakumar et al., 2010) and two distinct functionalization profiles deeply dependent on the specific PEOT/PBT ratio and polymer chain length (Table 1). Indeed, as indicated in Figure 2A, the UV irradiation of the PA 300 scaffold induced a photooxidation process capable of triggering an increase on the number of ester derivatives and carboxylic acids (288.7 eV) while the number of C-C = O bonds slightly declined (287.1 eV). Similarly, there was a practically imperceptible decrease on the number of C-OH and C-O-C functional groups (286 eV) due to the equilibrium between the production and consumption of oxygen moieties. The high percentage of PBT and the small polymer molecular weight were also decisive to avoid a significant amount of chain scission events (284.8 eV) during UV treatment. Contrary, after 40 min of UV irradiation, the PA 1000 electrospun network showed a fast and accentuated photo-oxidation process. This might be due to its longer and unordered PEOT macro-chains leading to easier diffusion of oxygen compared to the PA 300 scaffold, where increased crystalline segments could limit the access of oxygen more efficiently (Morlat and Gardette, 2001). This process is showed in Figure 2B, which reveals that the UV treatment of the PA 1000 fibrous system potentiated not only a noticeable number of polymer backbone chain fractures associated with the decrease of the C-C-C and C-H functionalities, but also a remarkable production of oxygenated functional groups (287.1 and 288.7 eV). Once again, the competition between the generation and consumption of C-OH and C-O-C functional groups did not enable a drastic change in the area of the C 1s peak located at 286 eV, showing only a marginal increase in the PA 1000 UV40 relatively to its untreated counterpart.

**Table 1:** Fractions of various functional groups from the C 1s peaks of the PA scaffolds.

**Scaffold**	**C 1s**			
	**284.8 eV**	**286 eV**	**287.1 eV**	**288.7 eV**
	**C-C-C; C-H**	**C-OH; C-O-C**	**C-C=O**	**O-C=O**
PA 300	63.6	25.1	7.9	3.3
PA 300 + UV 40	61.8	24.3	5.9	8
PA 1000	62.3	31.4	4	2.3
PA 1000 + UV40	47	36.1	7.6	9.3

**Figure 3:**
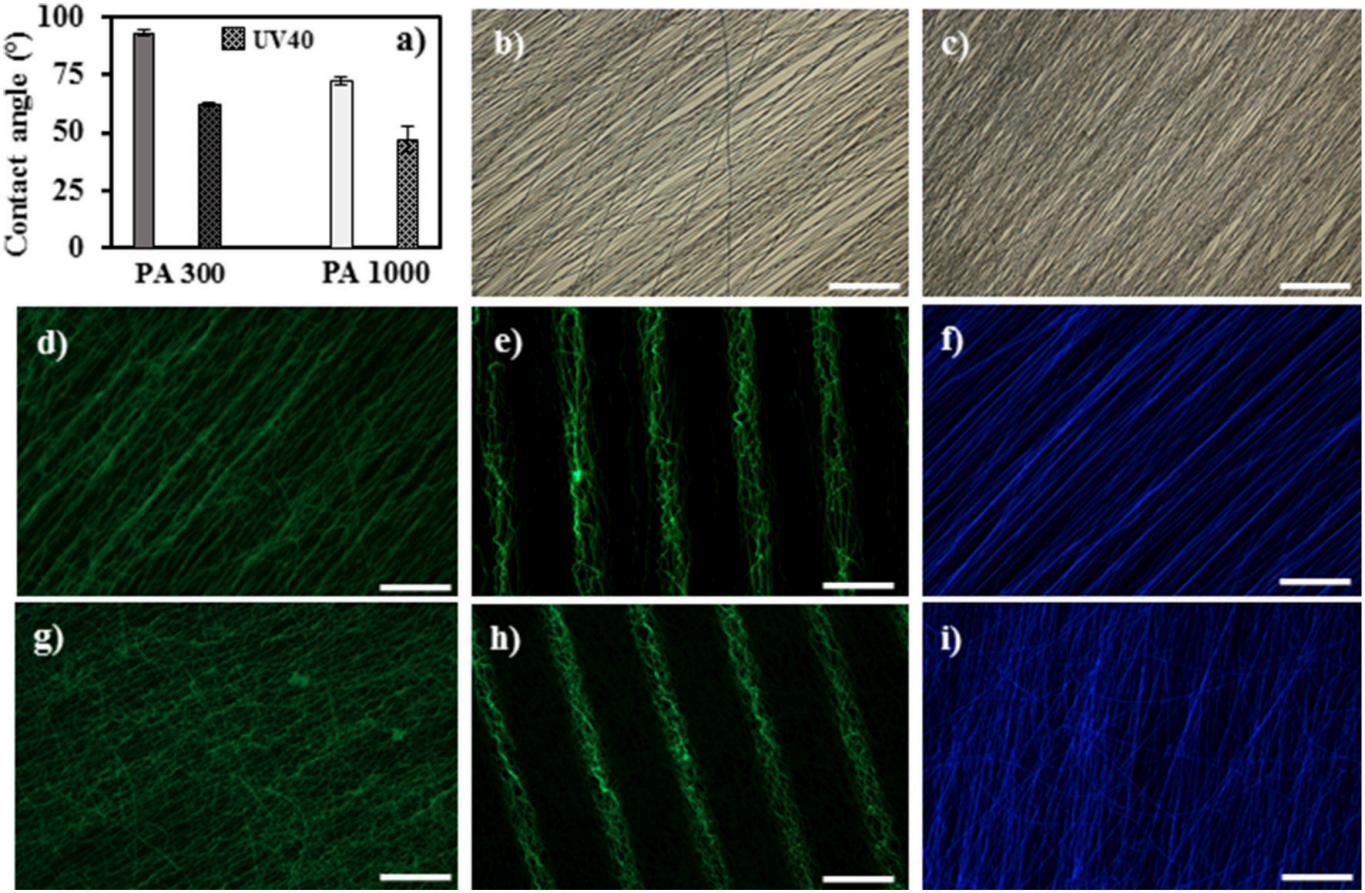
Wettability and protein adsorption tests of the PA scaffolds. **(a)** Contact angle on the PA materials before and after UV irradiation; bright field of the PA 300 UV40 + FITC-BSA **(b)** and PA 1000 UV40 + FITC-BSA **(c)** samples; green fluorescence **(d)**, photopatterning **(e)** and autofluorescence **(f)** of the PA 300 UV40 + FITC-BSA sample and green fluorescence **(g)**, photopatterning **(h)** and autofluorescence **(i)** of the PA 1000 UV40 + FITC-BSA sample. Scale bar = 100 μm.

The wettability variations triggered by the UV irradiation were analyzed via contact angle measurements, showing results that can be notoriously correlated with both ATR-FTIR and XPS analyses (Figure 3a). In detail, independently of the UV functionalization, the lower molecular weight of PEG and the higher content of PBT hydrophobic segments of the PA 300 polymer films have generated higher contact angles (92.8 ± 1.0 for untreated and 61.7 ± 0.6 for UV40) comparatively to the values measured for the PA 1000 films (72.3 ± 1.5 for untreated and 46.8 ± 5.4 for UV40). Moreover, the presence of a larger amount of oxygen functional groups after UV exposure encouraged an accentuated increase in the hydrophilicity of the PA 300 UV40 and PA 1000 UV40 materials relatively to their untreated counterparts (Papadaki et al., 2001; Olde Riekerink et al., 2003; Kutikov and Song, 2015). The main goal of the introduction of these oxygen moieties, particularly the carboxylic functional groups, onto the surface of the electrospun scaffolds was to enhance the immobilization of proteins and consequently modulate and improve cellular attachment. In fact, although the mechanisms behind protein adsorption are very complex (Rabe et al., 2011; Yu et al., 2015), several reports have highlighted the positive influence of COOH functionalities for anchoring proteins via either covalent bonding (Chen and Su, 2011; Zander et al., 2012) due to the use of coupling agents (e.g., carbodiimide hydrochloride/N-hydroxysuccinimide complex) or other non-specific binding (Meder et al., 2012; Nandakumar et al., 2012) governed by electrostatic forces, hydrogen bonding or hydrophobic interactions. Accordingly, the fluorescence imaging showed in Figure 3 confirms the ability of both PA 300 UV40 and PA 1000 UV40 to boost effective and homogeneous adsorption processes of FITC-BSA, contrasting to the untreated electrospun scaffolds where the protein immobilization was unsuccessful due to their poor oxygenated surfaces. Furthermore, it was possible to generate distinct biochemical cues onto the surfaces of the electrospun fibers by spatially controlling the interaction between the fibers and the UV radiation. As shown in Figures 3e,h, independently of the PEOT/PBT formulation, the pattern of intercalated active (green) and inactive (dark) zones precisely corresponded to the geometry of the used photomask, leading to the recreation of a biochemical pattern with an extremely accurate geometry. Collaterally, the UV irradiation induced strong blue autofluorescence in the PA 300 UV40 (Figure 3f) and PA 1000 UV40 (Figure 3i) samples. However, the phenomenon was not significant for the other wavelength fluorescence signals (red and green—data not shown). It is important to notice that both the autofluorescence and the uniform fluorescent protein distribution along the surfaces of the activated scaffolds could be depth dependent effects since it is unlikely that the porosities of the PA 300 UV40 and PA 1000 UV40 were able to prevent the loss of light intensity through thickness during UV exposure (Gestos et al., 2010).

**Figure 4:**
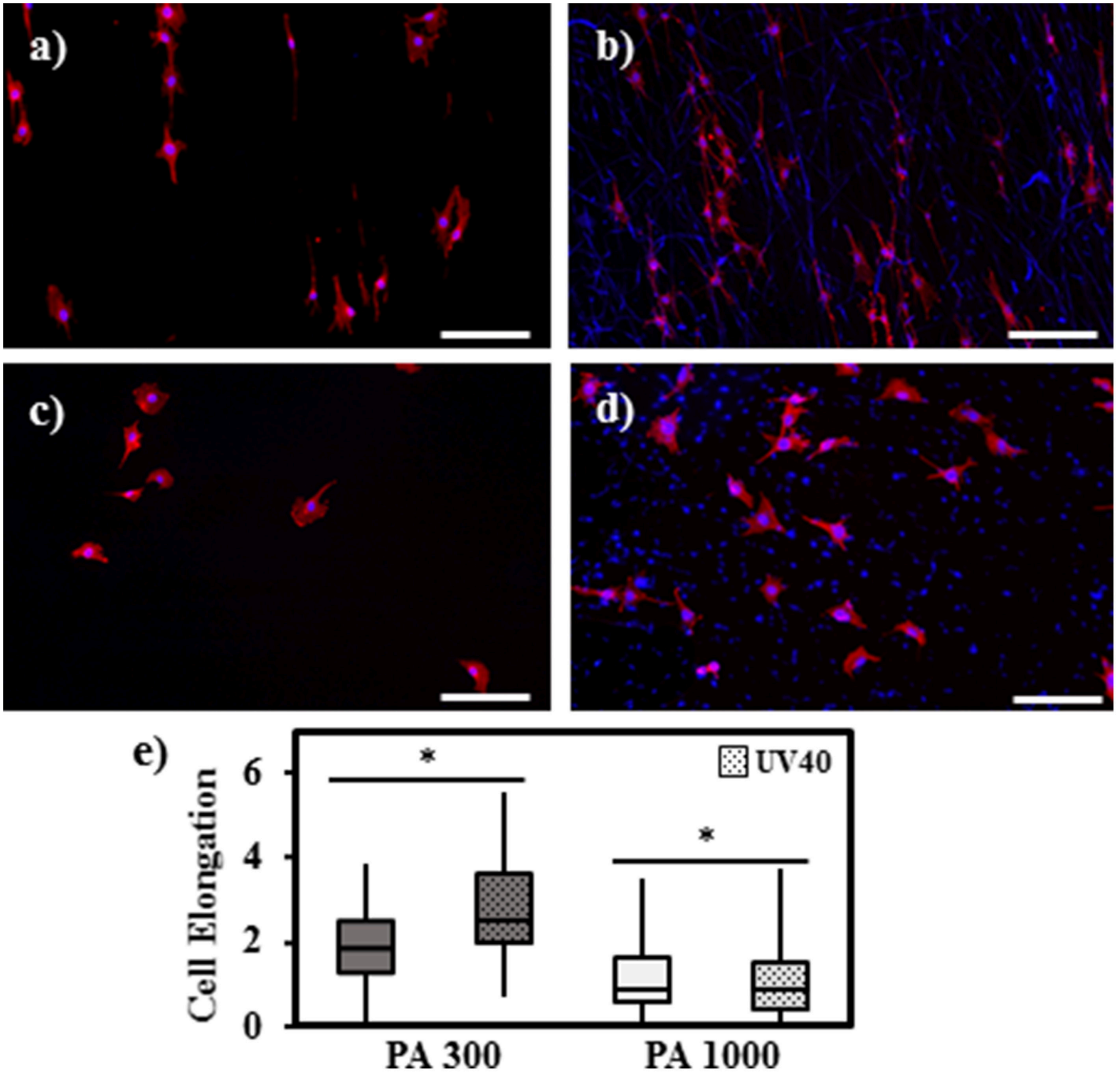
Cell adhesion and elongation on the PA scaffolds. **(a)** PA 300; **(b)** PA 300 + UV40; **(c)** PA 1000; **(d)** PA 1000 + UV40; **(e)** Cell elongation box diagram. Scale bar = 200 μm. ^*^indicates statistical significance (*p* < 0.05).

**Figure 5:**
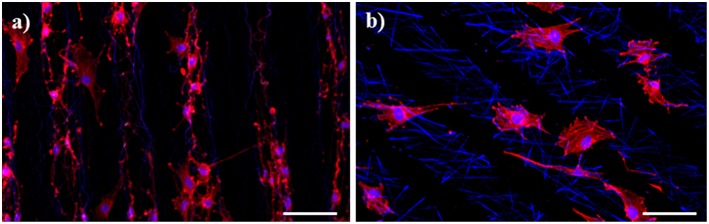
Cell adhesion on photopatterned PA 300 UV40 **(a)** and PA 1000 UV40 **(b)** scaffolds. Scale bar = 100 μm.

UV functionalization of the electrospun scaffolds also led to an increase in adsorbed serum proteins and therefore significantly improving cell adhesion and spreading compared to the untreated samples (Figure 4). Figures 4a,c show that the dissimilar surface properties of untreated PA 300 and PA 1000 promoted either a spindle-shaped phenotype or a flat morphology of the seeded rSCs, respectively. These morphological differences are analogous to the results reported in other studies (Papadaki et al., 2001; Hendriks et al., 2013; Kutikov and Song, 2015; Di Luca et al., 2016), whose authors have identified the hydrophobic level and the subsequent higher capacity to support protein adhesion of PA 300 as a critical factor for inducing complementary cell responses. The UV40 scaffolds (Figures 4b,d) did not induce any substantial variation on the morphology of the cells relatively to the untreated samples. However, it was possible to observe a larger number of attached rSCs, supporting the idea that the increasing number of functional groups available after UV exposure were critical for enhancing protein adsorption from the culture medium. In accordance with the fluorescence observations, the analysis of the elongation factor E revealed a statistically significant difference on the morphological features of the rSCs attached either onto PA 300 or PA 1000 electrospun fibers independently of UV exposure (Figure 4e). More precisely, the unaltered flattened morphology of the cells in both PA 1000 and PA 1000 UV40 scaffolds was translated into similar E values (1.11 ± 0.71 for PA 1000 and 1.13 ± 0.95 for PA 1000 UV40), contrasting with the more prominent elongation of the cells seeded onto PA 300 (E = 2.02 ± 1.00) and, particularly, onto PA 300 UV40 (E = 2.74 ± 1.18) electrospun meshes. The success of the UV induced biochemical pattern is evidently illustrated by Figure 5, where the cell adhesion and spreading occurred predominantly onto the active areas. Indeed, the effect of the photomask is particularly visible on the spatial distribution of the rSCs across the photopatterned PA 300 UV40 electrospun scaffold (Figure 5a), since they were not only successfully organized in parallel columns due to the UV functionalization, but also were able to elongate and promote lamellipodia formation in the direction of the fiber orientation. Contrary, although the rSCs showed to favor an attachment onto the active areas of the photopatterned PA 1000 UV40 (Figure 5b), their more rounded phenotype provoked a considerable colonization of the non-active fibrous regions.

Taking this into consideration, UV photopatterning proved to be a simple and efficient methodology to introduce biochemical gradients in both PA 300 and PA 1000 electrospun scaffolds, opening new design approaches to mimic complex microenvironments by spatially controlling the adhesion sites of cells without altering their morphology nor the topography of the fibrous networks.

## Conclusion

In this study, we revisited UV photopatterning with the purpose of easily generating biochemical gradients onto the surfaces of both PA 300 and PA 1000 electrospun scaffolds. Irrespective of the PEOT/PBT formulations and surface properties, it was possible to spatially introduce oxygen functional groups onto the fibrous networks and consequently customize the anchoring spots of the protein adhesion process. Such chemical modifications were able to enhance the adhesion of rSCs and specify the location of their attachment, leading to an optimized spreading along the active fibrous runways, particularly when PA 300 was used as bulk material. The presented results are suggestive of the potential of the UV irradiation, commonly used as sterilization procedure, to work as an affordable and suitable technique to immobilize biomolecules onto the surfaces of electrospun scaffolds, guaranteeing a complex and versatile range of combinations between the introduced biochemical gradients and the biomechanical cues provided by the electrospinning technique toward the design and fabrication of biomimetic TE platforms.

## Data Availability

All datasets generated for this study are included in the manuscript and/or the supplementary files.

## Author Contributions

PW, MA, RT, and LM conceptualized the study. AG performed the experiments. AG, PW, MA, SP, and PM analyzed and processed the data. AG, PW, MA, SM, and RvW wrote and reviewed the article.

### Conflict of Interest Statement

The authors declare that the research was conducted in the absence of any commercial or financial relationships that could be construed as a potential conflict of interest.
